# CHIP functions as an oncogene by promoting colorectal cancer metastasis via activation of MAPK and AKT signaling and suppression of E-cadherin

**DOI:** 10.1186/s12967-018-1540-5

**Published:** 2018-06-19

**Authors:** Jingjing Xu, Jun Zhou, Hanjue Dai, Fei Liu, Wenjing Li, Wenjuan Wang, Feng Guo

**Affiliations:** 1 Center for Clinical Laboratory, The First Affiliated Hospital of Soochow University, Suzhou, 215006 China; 2 Department of Oncology, The First Affiliated Hospital of Soochow University, Suzhou, 215006 China; 3 Department of Gastroenterology, The First Affiliated Hospital of Soochow University, Suzhou, 215006 China; 4 State Key Laboratory of Microbial Technology, School of Life Sciences, Shandong University, Jinan, 250012 China; 5 Center for Gene and Immunotherapy, The Second Hospital of Shandong University, Jinan, 250012 China; 6 Department of Oncology, Nanjing Medical University Affiliated Suzhou Hospital, Baita West Road 16, Suzhou, 215001 China

**Keywords:** CHIP, Colorectal cancer, Oncogene, Metastasis, Epithelial–mesenchymal transition

## Abstract

**Background:**

The carboxyl terminus of Hsc70-interacting protein (CHIP) is an E3 ubiquitin ligase that plays a controversial role in different cancers, either as a tumor suppressor or a tumor promoter. To date, the exact function and underlying mechanism of CHIP in colorectal cancer (CRC) is not yet clear. Here we aimed to determine whether CHIP could affect the biological behaviors of CRC cells and its underlying mechanisms.

**Methods:**

Stably transfected CHIP overexpression and depletion DLD-1 and HT-29 cells were established using Lipofectamine 2000. Cell growth was monitored by x-Celligence system. Cell proliferation was detected using CCK-8 and Brdu proliferation assay. Cell apoptosis and cell cycle were detected by flow cytometry analysis. Cell migration and invasion abilities were monitored by x-Celligence system, wound healing assay and transwell assay. In vivo intraperitoneal metastasis assay was performed to investigate the influence of CHIP on the tumor metastasis of CRC cells in nude mice. The expression of ERK, AKT, NF-кB signaling subunits and EMT related proteins were detected by Western blotting. The influence and function of CHIP on the protein expression of CRC cells were also elucidated by liquid chromatography–tandem mass spectrometry (LC–MS/MS) analysis. CRC microarray tissue was analyzed to investigate the CHIP expression and its clinical significance.

**Results:**

CHIP depletion inhibited cell growth, migration and invasion potential of CRC cells, accompanied by downregulation of MAPK and AKT signaling activities and upregulation of E-cadherin. CHIP overexpression dramatically enhanced the migration and invasion abilities, due to the upregulation of MAPK and AKT signaling and downregulation of E-cadherin. The proteomic analysis confirmed that E-cadherin was decreased in CHIP-overexpressing CRC cells. Furthermore, clinical tissue data revealed that CHIP expression was upregulated in CRC samples and was significantly correlated with poor survival of CRC patients. Mechanically, CHIP probably activated the MAPK and AKT signaling, which inactivated GSK-3β. The GSK-3β inactivation, in turn, upregulated Slug and led to E-cadherin downregulation and EMT initiation.

**Conclusions:**

Our finding suggested that CHIP functions as an oncogene in the migration and metastasis of CRC, and is a potential unfavorable independent predictive biomarker for CRC. CHIP activates the AKT pathway to promote EMT and metastasis in CRC through the CHIP-MAPK/AKT-GSK-3β-Slug-E-cadherin pathways.

**Electronic supplementary material:**

The online version of this article (10.1186/s12967-018-1540-5) contains supplementary material, which is available to authorized users.

## Background

Colorectal cancer (CRC) is the third most common cancer among both men and women worldwide, and is one of the leading causes of cancer-related deaths [1, 2]. With the change of dietary habits and lifestyle, the morbidity and mortality due to CRC have risen in Chinese population in the past few years. In 2015, the crude incidence and mortality rates of CRC in China both ranked 5th among all cancers [3]. The 5-year relative survival rate of CRC diagnosed without metastasis is 89.9%, but the outcome of patients with distant metastasis still remains unsatisfactory, with 5-year relative survival rate drops to 13.9%, despite of the consistent improvements in the development of more effective treatments [4, 5]. Therefore, more efforts are needed to elucidate of the underlying molecular mechanism of the CRC progression and metastasis, and to find the new effective therapeutic target for the metastatic CRC.

Epithelial–mesenchymal transition (EMT) is a process that is involved in embryonic development, wound healing, tissue remodeling, and also the malignant tumor metastasis [6, 7]. Epithelial cells are converted to mesenchymal phenotype, accompanied with a remarkable downregulation of epithelial markers, such as E-cadherin, cytokeratin 8/18 (CK8/18), and epithelial cell adhesion molecular (EpCAM), and an increased expression of mesenchymal-specific markers, such as N-cadherin, vimentin, and fibronectin [7, 8]. Epithelial cells adopt migratory and invasive properties in this process. E-cadherin, encoded by the *CDH1* gene, is the hallmark of EMT. It is a transmembrane glycoprotein, which is localized to adjacent cell membranes, and responsible for cell–cell interactions. Downregulation or loss of the E-cadherin, is reported to be involved in the invasion and metastatic progression of several malignancies, including CRC [9–12]. The expression of E-cadherin can be regulated by a variety of transcription factors, including Snail and Slug, which are belonging to the Snail family. Snail and Slug can bind to the promoter of the E-cadherin, and directly inhibit the transcription of the *CDH1* gene [13–17]. GSK-3β, a serine/threonine kinase, is inactivated through phosphorylation of the serine at residue 9 by the activation of AKT and mitogen-activated protein kinase (MAPK) signaling pathways [18–20]. Glycogen synthase kinase 3β (GSK-3β) could promote the phosphorylation and degradation of Slug, and subsequently trigger EMT and tumor metastasis [21].

The carboxyl terminus of the Hsc70-interacting protein (CHIP), also known as STIP1 homology and U-box containing protein 1 (STUB1), is a 34.5 kDa cytosolic protein. It is composed of a N-terminal tetratricopeptide repeat (TPR) domain that links to the chaperone heat shock protein 70/90 (Hsp70/90), a charged domain in the middle, and a C-terminal U-box domain that is essential for E3 ubiquitin ligase activity [22]. Mounting evidence reveal that CHIP can inhibit tumor proliferation, invasion, and progression in several malignancies, by regulating the ubiquitination and proteasomal degradation of a variety of oncogenic proteins, including TNF receptor-associated factor 2 (TRAF2) [23], nuclear factor kappa-light-chain-enhancer of activated B cells (NF-κB) [24], SRC-3 [25], receptor tyrosine-protein kinase erbB-2 (cErbB2/Neu) [26, 27], epidermal growth factor receptor (EGFR) [28], protein arginine methyltransferase 5 (PRMT5) [29], c-myc [30], and c-Met [31] and so on. However, other reports in glioma [32], esophageal squamous cell carcinoma (ESCC) [33], gallbladder carcinoma [34], and thyroid cancer [35] show opposite results about CHIP. Collectively, CHIP can function as an oncogene or a tumor suppressor, depending on its different targets [36]. To date, the exact function and underlying mechanism of CHIP in CRC have not been elucidated.

In the present study, we demonstrated that CHIP functioned as an oncogene and played a pivotal role in the metastasis of CRC. CHIP silencing suppressed the cell proliferation and inhibited cell growth, migration and invasion potential of CRC cells in vitro and in vivo, accompanied by downregulation of MAPK and AKT signaling activities and upregulation of E-cadherin. Although CHIP overexpression exerted little influence on cell growth of CRC cell lines, it dramatically promoted the migratory and invasive potential of CRC cells both in vitro and in vivo, due to the upregulation of MAPK and AKT signaling and downregulation of E-cadherin. The enhanced migratory and invasive abilities of CRC cells were predominantly contributed to the triggering of EMT via MAPK/AKT-GSK-3β-Slug-E-cadherin signaling. The proteomic analysis confirmed that E-cadherin was decreased in CHIP-overexpressing DLD-1 cells. We also found that CHIP was increased in CRC samples compared to that in paired adjacent non-neoplastic tissues. CHIP was correlated with worse clinical characteristics and poor survival and was a novel independent prognostic factor in CRC.

## Methods

### Cell culture and transfection

Human CRC cell lines HT-29, DLD-1, COLO320DM, and CaCO_2_ cells were purchased from the Shanghai Chinese Academy. Cells were grown in McCOY’s 5A, RPMI 1640 or Eagle’s minimum essential medium (EMEM) medium containing 10% FBS (Gibco, USA), 100 U/ml penicillin, 100 μg/ml streptomycin and 2 mM glutamine in a humidified incubator at 37 °C with an atmosphere of 5% CO_2_ according to the standard protocol. For CHIP silencing, CHIP shRNA was constructed on a backbone of pSilencer3.1-H1-neo vector. DLD-1 cells were transfected with pSilencer3.1-H1-neo (vector control) or pSilencer3.1-H1-neo-CHIP by use of Lipofectamine 2000 reagent (Cat Nr. 11668-019, Invitrogen, USA) according to the manufacturer’s instruction. The stable transfectants were then selected with G418 (400 μg/ml, Cat Nr. E-859, Amresco, USA).

CHIP overexpression plasmid was constructed on a backbone of MSCV vector containing GFP. 293-FT packaging cells were transfected with MSCV-GFP-CHIP or control vector (MSCV-GFP) by Lipofectamine 2000 reagent. Supernatants containing lentivirus particles were collected 48 h after transfection. DLD-1 and HT-29 cells were infected with the supernatants. Puromycin (5 μg/ml, Cat Nr. J593, Amresco, USA) was added to the culture medium to select the stable expressing cells.

### Clinical samples

The commercial CRC tissue microarray was obtained from Shanghai Outdo Biotech Company from the National Human Genetic Resources Sharing Service Platform (2005DKA21300). It consists of 87 paired CRC and non-neoplastic tissues and six other CRC samples only and were used to evaluate the expression of CHIP and E-cadherin protein. Patients’ follow-up information was obtained from 2009 to 2015.

### Immunohistochemistry

Paraffin-embedded tissues were deparaffinized in xylene, dehydrated in graded ethanol, rehydrated in water and subjected to 100 °C boiling in a 0.01 M citrate buffer (pH 6.0) for antigen retrieval. For IHC of cultured cells, cells cultured on glass slides for 48 h were fixed with 4% paraformaldehyde. Slides with tissues or cultured cells were then pretreated with 0.3% H_2_O_2_ to block the endogenous peroxidase activity and blocked with 1% bovine serum albumin to eliminate the nonspecific bindings. The sections were then incubated with the indicated primary antibodies at 4 °C overnight. After incubated with peroxidase-conjugated anti-rabbit/mouse secondary antibody, 3,3-diaminobenzidine (DAB) was used to visualize the expression of the indicated protein. Slides were observed by two independent investigators under an Olympus microscope BX51 (Olympus, Japan) and scored as follows: 0, No staining; 1+, light; 2+, moderate; 3+, strong, according to the intensity of the staining. The percentage of positive-stained cells was scored as follows: 0, no staining; 1, < 25% staining; 2, 26–50% staining; 3, 51–75% staining; and 4, > 75% staining. The product of the intensity and extent grades ≥ 4 of positive cells was considered high expression, and the score of 0–3 of positive cells was regarded as low expression.

### RNA isolation and quantitative real-time RT-PCR (qRT-PCR)

Cells were harvested, and total RNAs were extracted using TRIzol reagent (Cat Nr. 15596018, TaKaRa, Japan) according to the instruction manual and then quantified by NanoDrop 1000 (Thermo Fisher Scientific, China). cDNA was synthesized from 2 μg RNA by M-MLV reverse transcriptase (Cat Nr. 28025013, Promega, China) by the manufacturer’s instructions.

Quantitative real-time PCR was performed on LightCycler 480 instrument (Roche Diagnostics, China) with a 10-μl mixture composed of 2× LC480 SYBR-green IMaster Mix (Cat Nr. 11602920, Roche, USA), 500 nmol of each primer, and 300 ng of cDNA templates and ddH_2_O. The average relative mRNA expression levels of target genes were determined by the 2^−ΔΔCt^ algorithm, with *β*-*actin* as an internal control. The primers used are listed in Additional file 1. At least three independent experiments were performed for each single gene validation.

### Western blotting and antibodies

Harvested cells (10 × 10^6^) were lysed with a modified radioimmune precipitation assay (RIPA) buffer. The extract was quantified using a DC protein assay kit (Bio-Rad, USA). A 30-μg aliquot of protein was loaded and separated by SDS-PAGE and then transferred to nitrocellulose membranes. After nonspecific binding sites blocking, membranes were incubated with indicated primary antibodies at 4 °C overnight. Subsequently, membranes were probed with an appropriate secondary antibody. The specially bound antibodies were detected and scanned using an Odyssey system (LI-COR Biosciences, Lincoln, NE, USA). Abs against RelA (sc-372X), RelB (sc-226X), p105/p50 (sc-7178X), p100/p52 (sc-298) and c-Rel (sc-70) were purchased from Santa Cruz Biotechnology. Abs against CHIP (#2080) ERK1/2 (#4695), p-ERK1/2 (#4370S), AKT (#4691), p-AKT^308^ (#2965), p-AKT^473^ (#4060), cyclinD1 (#2978), GSK-3β (#12456), p-GSK-3β (#5558), E-cadherin (#3195), EpCAM (#14452), CK8/18 (#4546) and Slug (#9585) were obtained from Cell Signaling Technology. Actin (A01215a) was obtained from Abgent. IRDye 680CW (#926-32222) and IRDye 800CW secondary Abs (#926-32210) were obtained from LI-COR Biosciences.

### Real-time cell growth assay

Cell growth was performed with a dynamically real-time x-Celligence RTCA monitoring system (Roche Diagnostics, China). In this assay, 10,000 cells/well were seeded, resuspended in 200 μl culture media (supplemented with 10% FBS) on the E-plate. Cell impedance was continuously monitored for 72 h, and the value was measured as ‘cell index’ according to the cells number, cells size and morphology, and the interaction degree of the cells to the sensor surface. Data were collected and analyzed by RTCA software 1.2.

### BrdU proliferation assay

The BrdU Cell Proliferation Assay Kit (Cat Nr. 2750, Merck Millipore, German) was used to detect the proliferation of CRC cells according to the manufacture’s instruction. The relative number of actively proliferating cells was determined by measuring the amount of BrdU incorporation. BrdU label was added to the cultured cells, incubated for 4 h at 37 °C before detection. After incubated with anti-BrdU monoclonal for 1 h and Goat anti-mouse IgG peroxidase conjugate for 30 min, the Substrate was added, and the reaction was stopped 30 min later. Finally, optical density (OD) at 450/550 nm was measured using spectrophotometer microplate reader (Biotek, USA). Each data point is the average of six determinations and each experiment was repeated in triplicate.

### Apoptosis evaluation

Cells were harvested at each indicated time point of 24, 48, and 72 h, washed twice with cold PBS, and stained with AnnexinV-APC and PI using APC-AnnexinV Binding apoptosis assay kit (Cat Nr. 22837, AAT Bioquest, USA) for 15 min at room temperature according to the manufacturer’s instructions (Invitrogen, China). Apoptosis cells were quantified using FACSCalibur™ cytometer (BD Biosciences, USA), and analyzed by CellQuestPro software.

### Cell cycle analysis

The different phases of the cell cycle were determined by flow cytometry analysis. Cells were harvested and fixed with 70% ethanol for 24 h at 4 °C. 50 μg/ml PI (Cat Nr. P4170, Sigma, German) and RNase A (Cat Nr. 12091-021, invitrogen, USA) were added to the single cell suspension and incubated for 30 min at 37 °C according to the manufacturer’s instructions. Cellular DNA content measurement was carried out using FACSCalibur™ cytometer, with three independent experiments displayed. The percentage of the cell population in each cell cycle phases, including G0/G1, S and G2/M were calculated according to the DNA content histograms.

### Cell migration and invasion assays

#### Real-time cell migration assay

In this assay, CIM-plate assembled with upper and lower chamber was used to determine the cell migration with the x-Celligence RTCA instrument. RPMI-1640 media (170 μl) supplemented with 10% FBS was added in each well on the lower chamber. 40,000 cells resuspended in 100 μl culture media without serum were seeded in each well on the upper chamber. After attachment, cell migration towards lower chamber was continuously monitored for 24 h, and data were collected and analyzed by RTCA software 1.2.

#### Wound healing assay

Cells were seeded into six-well plates and reached to 80% confluence for 24 h. The monolayer was scratched with a sterile 200 μl-micropipette tip and washed with PBS for three times to rinse off the detached cells. Cells were then cultured with RPMI-1640 supplemented with 10% FBS for further 72 h. The wound closure was observed and photographed at 0, 24, and 48 h, under the Light System Microscope IX71 (Olympus, Japan).

#### Transwell assay

The cell migration and invasion potential were assessed using 6.5-mm Transwell chambers (Falcon cell culture inserts, 8-μm pore size, BD Biosciences, USA) according to the manufacture protocol provided. Briefly, 40,000 cell were resuspended in 150 μl serum-free media and were seeded into each upper chamber with the lower chamber filled with 600 μl of complete medium (supplemented with 10% FBS) and incubated for additional 24 h. For invasion assay, the transwell insert was pre-coated with 50 μl Matrigel (Cat Nr. 354234, BD Bioscience, USA) and dried in 37 °C. After 24 h, the cells on the upper surface of the insert were removed slightly with a cotton swab, the cells attached to the lower surface of the insert were migrating or invasive cells. The cells were fixed with methanol for 20 min, and then stained with 0.1% of crystal violet for 30 min and washed with PBS for at least three times, and then photographed under a microscope. Each experiment was displayed in three independent replicate. Cells were counted in five randomly selected fields, and the average migrated and invasive cell number was calculated.

#### In vivo intraperitoneal metastasis assay

For the in vivo intraperitoneal metastasis assay, cells were cultured and harvested. 6-week old BALB/c male mice (Shanghai Experimental Animal Company, China) were divided into four groups, group siCHIP, sictrl, hCHIP, and ctrl, randomly. Each mouse was given an intraperitoneal injection with indicated 5 × 10^6^ DLD-1 transfected cells which suspended in 100 μl PBS. Mice were housed under sterile conditions during all experimentations on a 12-h light–dark cycle and were sacrificed after 6 weeks. The number of metastatic foci on the mesentery surface was counted. The tumors were then immediately fixed in 4% paraformaldehyde and embedded in paraffin (FFPE tissues) for subsequent histological analysis of CHIP, or collected for the preparation of whole cell extract. The study was approved by the Joint Ethics Committee of Animal Experiments of the First Affiliated Hospital of Soochow University.

### Liquid chromatography–tandem mass spectrometry (LC–MS/MS) analysis

Whole cell extracts were lysed in extraction buffer (8 M urea, 0.1% sodium dodecyl sulfate) containing additional 1 mM PMSF and protease inhibitor cocktail on ice for 30 min and quantified with BCA assay. 100 μg total protein was selected, and isobaric tags for relative and absolute quantification (iTRAQ) labels were performed according to the manufacturer’s instructions (AB Sciex Inc., USA) at CapitalBio Technology, Beijing. The LC–MS/MS analysis was carried out in CapitalBio Technology using a Q Exactive mass spectrometer (Thermo Scientific, USA). In this study, a protein was considered differentially expressed when it had a fold change of > 1.5.

### Statistical analysis

The data were presented as mean ± standard deviation (*SD*). Data were analyzed by Student’s t test between two groups. The χ^2^ test was used to exam the differences in the distributions between the variables. The survival curves were plotted using Kaplan–Meier analysis and compared by log-rank test. The association between CHIP and E-cadherin protein expression was analyzed by Spearman’s test (r; *P* value). Multivariate Cox regression analysis was also performed. A *P* value < 0.05 was considered as statistically significant. Statistical analysis was performed using SPSS 24. Figures were created using GraphPad Prism 5.0.

## Results

### CHIP silencing inhibits cell growth of DLD-1 cells

To evaluate the expression of CHIP in CRC cell lines, qRT-PCR and western blot analysis were carried out. As shown in Additional file 2a, the *CHIP* mRNA expression of DLD-1 and COLO320DM cells was at a remarkable high level compared to that of HT-29 and CaCO2 cell lines. Western blotting analysis showed that the CHIP protein expression was much higher in the HT-29 and DLD-1 cells compared to that in the COLO320DM and CaCO_2_ cells (see Additional file 2b). Hence, the DLD-1 and HT-29 cell lines with abundant CHIP protein expression were chosen to perform the following experiments.

To elucidate the function of CHIP in CRC cells, DLD-1 cells were transfected with either a recombinant pSilencer3.1-H1-neo-CHIP shRNA or pSilencer3.1-H1-neo shRNA (control) plasmid, respectively. Stably transfected cell clones were selected using G418 at a concentration of 400 ng/μl. RNA interference of *CHIP* was successfully established, indicated by clear reduction of *CHIP* expression both at mRNA (see Additional file 3a) and protein level (see Additional file 3b) in siCHIP cells.

**Fig. 1:**
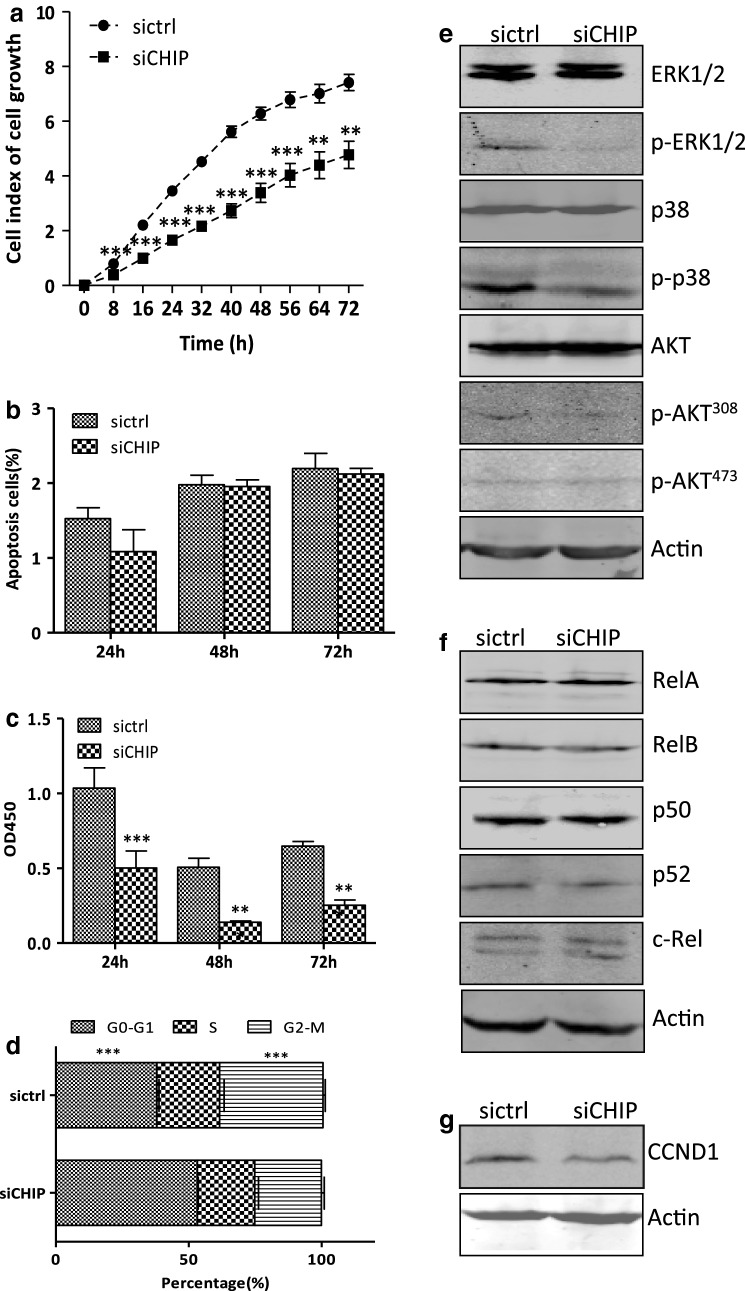
CHIP silencing inhibits cell growth of DLD-1 cells. **a** The cell growth curves of the siCHIP and sictrl cells were detected using a real-time x-Celligence system. E-plate was plated with 10,000 cells/well and the cell growth was continuous monitored for 72 h. Significant differences were indicated (Student’s t test, **P < 0.01, ***P < 0.001). **b** The apoptosis of the siCHIP and sictrl cells was determined by the AnnexinV/PI staining. AnnexinV positive and AnnexinV/PI positive cells were determined as apoptosis cells. Values ± *SD* from three individual experiments were displayed. **c** Cell proliferation of the siCHIP and sictrl cells was determined by the Brdu proliferation assay. 96-well plate was plated with 10,000 cells/well and was added 10 μl buffer after cultured for 24, 48 and 72 h. OD450 was measured using spectrophotometer microplate reader. Significant differences were indicated (Student’s t test,***P < 0.001). **d** CHIP depletion caused cell cycle arrest at the G0–G1 phase. Cell cycle parameters of siCHIP and sictrl cells were determined by flow cytometry analysis using propidium iodide. The histograms report the percentage of cells in G0/G1, S and G2/M phases of all the establishing cell lines. ***P < 0.001. **e** Western blotting analysis of the protein expression of total ERK1/2, p-ERK1/2, p38, p-p38, as well as total AKT andp-AKT^473^ and p-AKT^308^ in siCHIP and sictrl cells. Actin was used as an internal control. **f** Western blotting analysis of the protein expression of the NF-κB subunits. Actin was used as an internal control. **g** Western blotting analysis of the protein expression of the cell cycle-related protein cyclinD1. Actin was used as an internal control

Cell growth of siCHIP and sictrl cells was monitored continuously using a real-time x-Celligence system for 72 h. As shown by the cell growth curve in Fig. 1a, the siCHIP cells grew much slower than the sictrl cells, and had statistical significances at all the indicated time points (**P < 0.01, ***P < 0.001). The spontaneous apoptosis rarely occurred in both cell lines, and had no statistical significances at 24, 48, and 72 h time points (Fig. 1b). The OD450 values, detected in Brdu proliferation assay, were 0.50 ± 0.11, 0.14 ± 0.01, 0.25 ± 0.04 in the siCHIP cells, and 1.04 ± 0.13, 0.51 ± 0.06, 0.65 ± 0.03 in the sictrl cells at 24, 48, and 72 h (*P *< 0.001). Thus, the CHIP silencing significantly decreased the DLD-1 cell proliferation capability (Fig. 1c). Therefore, the CHIP silencing suppressed DLD-1 cell growth in vitro, likely owing to the impaired cell proliferation.

The effect of CHIP expression on cell cycle regulation in DLD-1 cells was measured by flow cytometry analysis. The percentages of the siCHIP cells in three cell cycle phases (G0/G1, S, and G2/M) were 53.21 ± 0.38, 21.59 ± 1.45 and 25.10 ± 1.07%, while those of the sictrl cells were 38.02 ± 0.80, 23.61 ± 1.70 and 38.90 ± 0.86%, respectively. Thus, CHIP silencing significantly induced a strong accumulation of the cells in G0-G1 phase compared to that in the sictrl cells (*P *< 0.001). The percentage of siCHIP cells in G2/M was decreased by about 13% (*P *< 0.001) (Fig. 1d). CHIP silencing suppressed the expression of cyclinD1 in DLD-1 cells (Fig. 1g).

As shown in Fig. 1e, the phosphorylation of ERK1/2 (p-ERK1/2) as well as phosphorylation of p38 (p-p38) was markedly reduced in the siCHIP cells, compared to those in the sictrl cells. Total ERK1/2 and p38 expression were unchanged in both cell lines. The phosphorylation of AKT on threonine 308 (p-AKT^308^) was also obviously decreased in siCHIP cells, compared to that in the sictrl cells. The expression of total AKT and the phosphorylation of AKT on serine 473 (p-AKT^473^) were unchanged in both cell lines. The expression of RelA/p65 and p50 (representing the canonical NF-κB activities), and the expression of RelB and p52 (representing the non-canonical NF-κB activities) were similar in the siCHIP and sictrl cells (Fig. 1f). CHIP silencing exerted little effects on the NF-κB signaling pathway.

Taken together, the results here indicated that the expression of CHIP influenced cell proliferation and G0-G1 transition in DLD-1 cells. The inactivated MAPK and AKT signaling pathways, along with the downregulated cell cycle-related protein contributed to the suppressed cell proliferation and G0–G1 arrest in the absence of CHIP.

### CHIP overexpression did not affect cell growth of DLD-1 cells

To establish a CHIP-overexpressing DLD-1 cell line, we stably transfected reconstructed plasmid carrying the human *CHIP* cDNA (MSCV-GFP-CHIP) or control vector (MSCV-GFP) into the DLD-1 cells. Transfected cells were cultured in the presence of puromycin (5 ng/μl) to get monoclonal cells. The cells were further verified for the CHIP expression both at mRNA and protein level by qRT-PCR and western blotting analysis. As shown, *CHIP* was remarkably induced in hCHIP cells (the cells transfected with MSCV-GFP-*CHIP*) both at mRNA (see Additional file 3c) and protein levels (see Additional file 3d), compared to that in ctrl cells (the cells transfected with MSCV-GFP). Therefore, DLD-1 cell line overexpressing the human *CHIP* cDNA was successfully established.

**Fig. 2:**
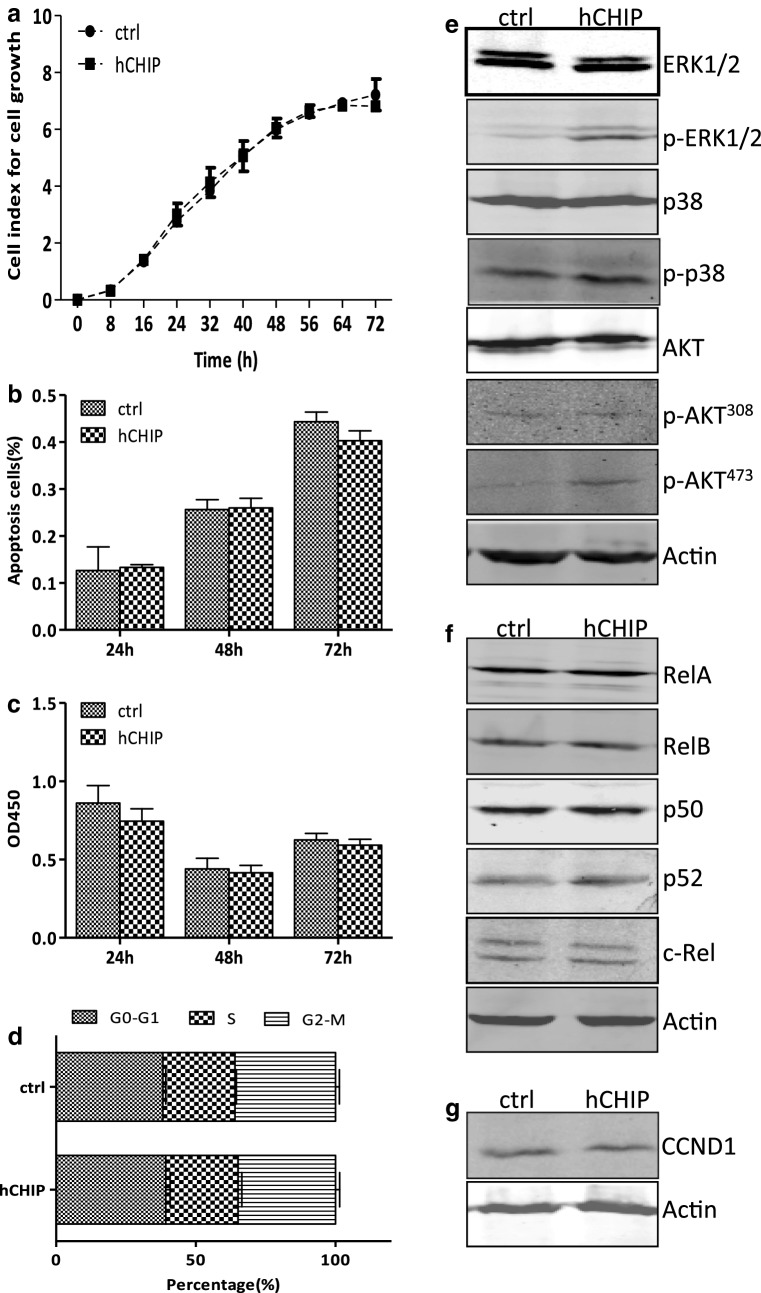
CHIP overexpression did not affect cell growth of DLD-1 cells. **a** The cell growth curves of the hCHIP and ctrl cells were detected using a real-time x-Celligence system. E-plate was plated with 10,000 cells/well and the cell growth was continuous monitored for 72 h. **b** The apoptosis of the hCHIP and ctrl cells was determined by the AnnexinV/PI staining. AnnexinV positive and AnnexinV/PI positive cells were determined as apoptosis cells. Values ± SD from three individual experiments were displayed. **c** Cell proliferation of the hCHIP and ctrl cells were determined by the Brdu proliferation assay. 96-well plate was plated with 10,000 cells/well and was added 10 μl buffer after cultured for 24, 48 and 72 h. OD450 was measured by means of spectrophotometer microplate reader. **d** Cell cycle parameters of hCHIP and ctrl cells were determined by flow cytometry analysis using propidium iodide. The histograms report the percentage of cells in G0/G1, S and G2/M phases of all the establishing cell lines. **e** Western blot analysis of the protein expression of total ERK1/2, p-ERK1/2, p38, p-p38 as well as total AKT and p-AKT^473^ and p-AKT^308^ in hCHIP and ctrl cells. Actin was used as an internal control. **f** Western blot analysis of the protein expression of the NF-κB subunits. Actin was used as an internal control. **g** Western blot analysis of the protein expression of the cell cycle related proteins cyclinD1. Actin was used as an internal control

To investigate whether CHIP overexpression affected the cell growth of DLD-1 cells, we monitored continuously cell growth of hCHIP and ctrl cells by a real-time x-Celligence system for 72 h. There were no obvious differences between the hCHIP and the ctrl cells for all the indicated time points (Fig. 2a). The spontaneous apoptosis rarely occurred in both hCHIP and ctrl cells, with no statistical significances at 24, 48 and 72 h time points (*P *> 0.05) (Fig. 2b). Cell proliferation assay was further performed to evaluate the cell growth of the two establishing cell lines. As shown in Fig. 2c, the OD450 values, detected in Brdu proliferation assay, were comparable at all the indicated time points (*P *> 0.05). The OD450 values were 0.75 ± 0.08, 0.42 ± 0.05, 0.59 ± 0.04 in the hCHIP cells, and 0.86 ± 0.11, 0.44 ± 0.07, 0.63 ± 0.04 in the ctrl cells at 24, 48, and 72 h, respectively.

To find whether CHIP plays a similar role in other CRC cells, we performed the same experiments using HT-29 cells. As shown in Additional file 4a, b, CHIP expression was evidently induced in HT-29 hCHIP cells compared to that of ctrl cells. Similar to DLD-1 hCHIP cells, there was no obvious difference in cell growth between the HT-29 hCHIP and the ctrl cells, monitored by the x-Celligence system (see Additional file 4c). The Brdu proliferation assay indicated that the cell proliferation of hCHIP and ctrl cells were comparable at all the indicated time (see Additional file 4d).

There were no statistical significances between the percentage of the cell cycle phases from the hCHIP and the ctrl groups (*P *> 0.05) (Fig. 2d). The percentages of the hCHIP cells in G0/G1, S and G2/M phases were 39.25 ± 1.57, 25.91 ± 1.32 and 34.84 ± 1.48%, while those of the ctrl cells were 38.26 ± 0.96, 25.74 ± 0.48 and 36.00 ± 1.42%, respectively. CHIP overexpression exerted no influence on the expression of cyclinD1 in DLD-1 cells (Fig. 2g).

As shown in Fig. 2e, the protein expression of p-ERK1/2 and p-p38 were markedly induced in hCHIP cells when compared to those in ctrl cells. While the expression of total ERK1/2 and p38 were comparable to those in ctrl cells. The expression of p-AKT^473^ was markedly induced in hCHIP cells when compared to that in ctrl cells. While the expression of total AKT and p-AKT^308^ were comparable to those in ctrl cells. There were no obvious changes in the protein expression of all the NF-κB subunits (Fig. 2f). Therefore, CHIP overexpression did not affect the NF-κB signaling activities.

### CHIP promotes the migration and invasion abilities of DLD-1 Cells in vitro and in vivo

**Fig. 3:**
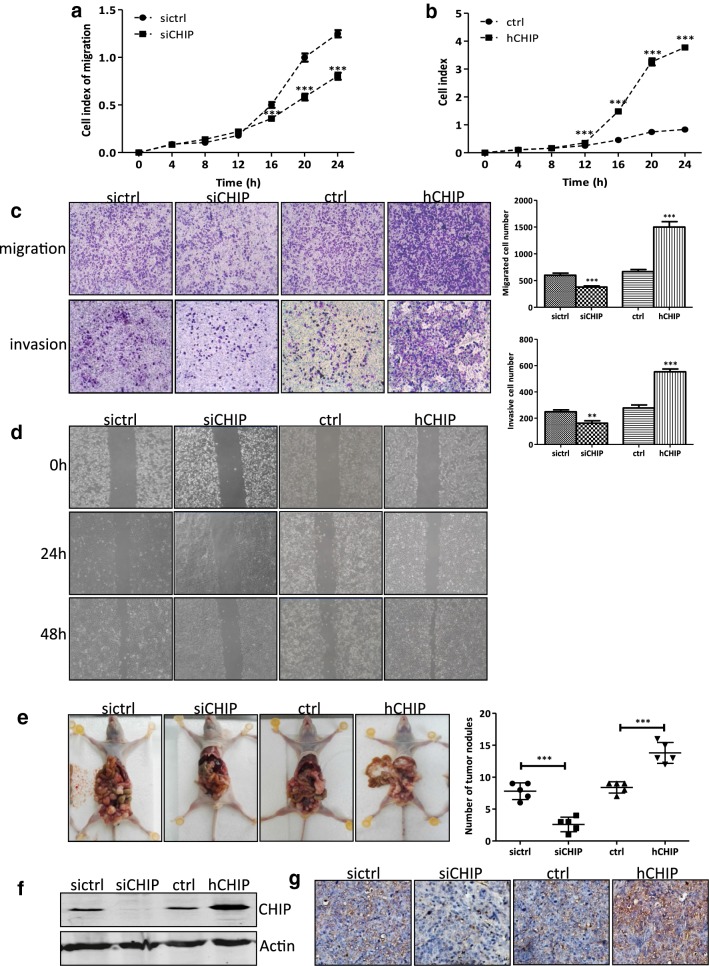
CHIP promotes the migration and invasion abilities of DLD-1 cells in vitro and in vivo. **a**, **b** The cell migration curves between the siCHIP and sictrl cells (**a**) as well as hCHIP and ctrl cells (**b**) were detected using a real-time x-Celligence system. CIM-plate was seeded with 40,000 cells/well and the cell migration was continuous monitored for 24 h. Significant differences were indicated (Student’s t test,***P < 0.001). **c** Representative images and data of a Transwell migration and invasion assay for the establishing DLD-1 cell lines. The siCHIP and sictrl cells, as well as hCHIP and ctrl cells, were allowed to migrate through the transwell inserts [pre-coated with Matrigel (1:40) or not] for 24 h. Upper inserts were seeded with 40,000 cells/well in 150 μl RPMI-1640 media without serum. The lower chambers were filled with 600 μl RPMI-1640 media supplemented with 10% FBS. The number of migrated and invasive cells were fixed, stained, photographed, and compared with the control group. Each bar represents the mean ± SD. **P < 0.01, ***P < 0.001. All images were representative of at least three independent experiments with similar findings. **d** Would healing assay for the siCHIP and sictrl cells as well as hCHIP and ctrl cells. Confluent monolayers of the indicated cells were scraped with a pipette tip to generate wounds and then were cultured for 48 h. Representative images of wounds were recorded with the Light System Microscope IX71 at 0, 24, and 48 h. All images are representative of at least three independent experiments with similar findings. **e** Assessment of metastatic capacity of 5 × 10^6^ DLD-1 cells stably expressing siCHIP, hCHIP and each control cells by inoculating nude mice via intraperitoneal injection. 6 weeks later, the mice were sacrificed, and metastatic nodules on the mesentery were collected. The numbers of the metastatic nodules were measured (***P < 0.001). **f** Whole cell extract of the metastatic nodules was prepared. The protein expression of CHIP was determined by western blotting. Actin was used as a loading control. **g** IHC analysis of CHIP expression in representative metastatic nodules (×200 magnification)

To investigate whether CHIP plays a role in cell migration abilities of DLD-1 cells, cell migration of the siCHIP and sictrl cells, as well as the hCHIP and ctrl cells, was monitored continuously by a real-time x-Celligence system for 24 h (Fig. 3a, b). Knockdown of CHIP expression significantly impaired the migratory potential of DLD-1 cells in a time-dependent manner, and had a statistically significant difference from 16 to 24 h (*P *< 0.001, Fig. 3a). In contrast, CHIP overexpression dramatically increased the migration ability of DLD-1 cells in a time-dependent manner and had a statistically significant difference from 12 to 24 h (*P *< 0.001, Fig. 3b). Transwell chambers were also used to examine the role of CHIP on the in vitro migration abilities of DLD-1 cells. As shown in Fig. 3c, the number of migrated siCHIP cells was significantly lesser than that of the sictrl cells (*P *< 0.001), while the number of migrated hCHIP cells was notably greater than that of the ctrl cells (*P *< 0.001). Consistently, the CHIP silencing significantly hampered the wound closure of DLD-1 cells, while the CHIP overexpression promoted the wound closure as compared to the ctrl group, indicating the enhanced migration potential in hCHIP cells (Fig. 3d).

Transwell inserts pre-coated with Matrigel were used to assess whether the CHIP expression affects the cell invasion in vitro. As shown in Fig. 3c, the number of invaded siCHIP cells was significantly lesser than that of the sictrl cells (*P *< 0.01), while the number of invaded hCHIP cells was greater than that of the ctrl cells (*P *< 0.001).

In line with the findings in the DLD-1 cells, the migratory and invasive potential of CHIP-overexpressing HT-29 cells were significantly enhanced compared with those of the ctrl cells (*P *< 0.001) (see Additional file 4e, f).

To further investigate both the gain-of-function and the loss-of-function of CHIP in DLD-1 cells on tumor metastasis in vivo, the nude mice were intraperitoneally injected with siCHIP/sictrl cells as well as hCHIP/ctrl cells. 6 weeks after injection, overexpression of CHIP enhanced the mesentery metastasis in nude mice. More metastatic nodules on the mesentery were formed in mice injected with the hCHIP cells compared with those injected with the ctrl cells (*P *< 0.001). On the contrary, less metastasis nodules were formed on the mesentery in mice injected with the siCHIP cells than those injected with the sictrl cells (*P *< 0.001, Fig. 3e).

Western blotting analysis of the metastasis nodules revealed that CHIP protein was substantially downregulated in the siCHIP group compared with that in the sictrl group. Whereas tumor tissues from the mice injected with the hCHIP cells had upregulation of CHIP expression compared with that from the ctrl group (Fig. 3f). IHC examination exhibited the similar results, and the representative images of CHIP expression were shown in Fig. 3g. Taken together, these results suggested that CHIP positively regulated metastasis of the DLD-1 cells both in vitro and in vivo.

### CHIP triggers the EMT of DLD-1 cells via inactivating GSK-3β

**Fig. 4:**
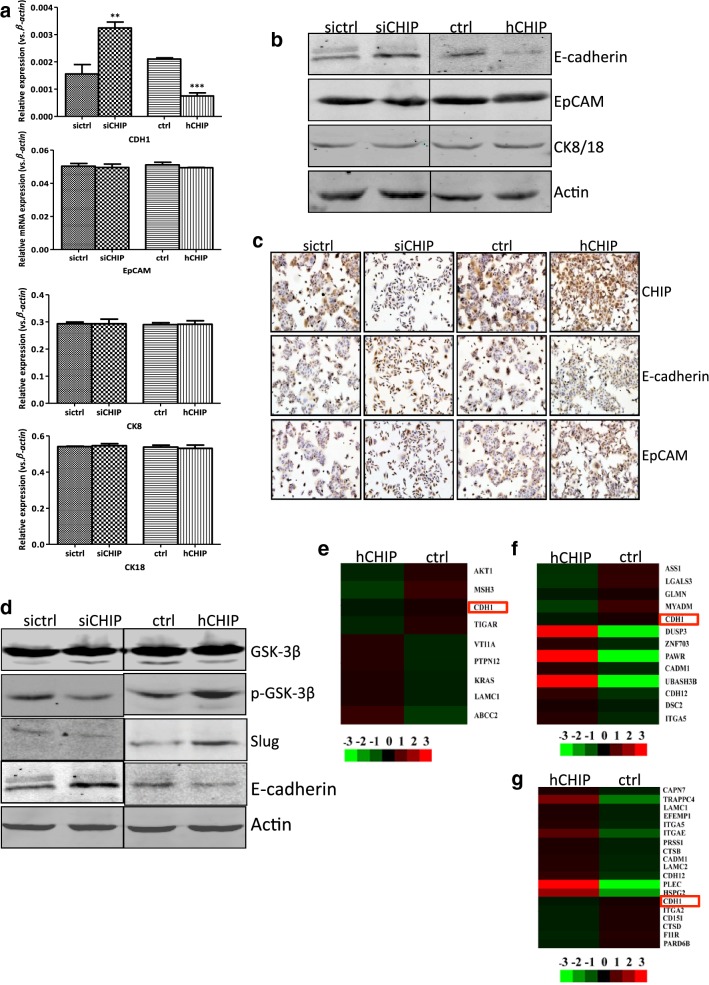
CHIP triggers the EMT of DLD-1 cells via inactivating GSK-3β. **a** The mRNA expression of *CDH1*, *EpCAM*, *CK8*, and *CK18* in the established DLD-1 transfected cell lines, including siCHIP and sictrl, as well as hCHIP and ctrl cells. *β*-*actin* normalized gene expression, measured in triplicates was displayed. Significant differences were indicated (Student’s t test, **P < 0.01, ***P < 0.001). **b** The protein expression of E-cadherin, EpCAM, and CK8/18 in established DLD-1 transfected cell lines were analyzed by western blotting. The level of each protein was normalized against Actin. **c** Representative IHC images for CHIP, E-cadherin, and EpCAM expression in the established DLD-1 transfected cell lines (×200 magnification). **d** Western blotting analysis of the protein expression of GSK-3β, p-GSK-3β, Slug, and E-cadherin in the DLD-1 established transfected cell lines. The level of each protein was normalized against Actin. **e**–**g** Hierarchical clustering analysis on the differentially expressed proteins among DLD-1 CHIP-overexpressing and control cells, which related to the CRC disease and cell–cell adhesion and junction based on the disease, GO, and KEGG pathway enrichment analysis, respectively. CDH1 is marked by a red box

EMT confers migratory and invasive properties to cancer cells and plays a fundamental role in the progression and metastasis in carcinoma. During the procession of EMT, the biomarkers were often converted from epithelial-characteristic to mesenchymal-characteristic. As shown in Fig. 4a, b, CHIP silencing upregulated the protein level of E-cadherin in the DLD-1 cells that was in line with the upregulation of *CDH1* at mRNA level.

In contrast, the E-cadherin expression was decreased in the CHIP-overexpressing cells both at mRNA and protein level compared to that in the ctrl cells. IHC staining of certain EMT markers in individual cell lines was performed. Similarly, the expression of E-Cadherin was reduced in the hCHIP cells, while the expression of E-cadherin was induced in the hCHIP cells (Fig. 4c). Other epithelial markers, such as EpCAM and CK8/18, were not affected by either loss-of-function or gain-of-function of CHIP in DLD-1 cells, both at mRNA level and protein level detected by Western blotting analysis and IHC staining (Fig. 4a–c). Thus, it is possible that CHIP regulated the expression of E-cadherin in DLD-1 cells, and subsequently triggered the occurrence of EMT.

E-cadherin can be directly modulated by a variety of transcription factors including Snail, Slug, and others [13, 14]. GSK-3β, inactivated by the MAPK and AKT signaling pathway, promotes the degradation of Slug and subsequently triggers EMT in lung cancer [21]. In the present study, we found that the CHIP overexpression activated the MAPK and AKT signaling in the DLD-1 cells. Thus, we wondered whether CHIP influenced the activity of GSK-3β. The diminished phosphorylation of Ser9, leading to GSK-3β activation, was observed in the siCHIP cells. Conversely, the increased phosphorylation of Ser9, leading to GSK-3β inactivation, was observed in hCHIP cells compared to that in the ctrl cells. The expression of Slug was negatively correlated with the GSK-3β activity (Fig. 4d).

Comprehensive proteomic analysis by LC–MS/MS analysis was performed to compare the protein signature between the hCHIP cells and ctrl cells. 790 proteins with a cutoff value of 1.5-fold change were differentially expressed in CHIP-overexpressing cells when compared to that in ctrl cells, including 414 upregulated proteins and 376 downregulated proteins. E-cadherin expression was confirmed to be downregulated in the hCHIP cells by the LC–MS/MS analysis. According to the disease enrichment analysis, 9 out of the 790 differentially expressed proteins were correlated to the CRC disease, including E-cadherin (Fig. 4e). Gene ontology (GO) enrichment analysis (Fig. 4f) and kyoto encyclopedia of gene and genomes (KEGG) pathway analysis (Fig. 4g) also revealed that the downregulation of E-cadherin was also correlated to the cell–cell adhesion and cell–cell junction pathway, respectively.

### Clinical significance of CHIP and E-cadherin expression in CRC tissues

**Fig. 5:**
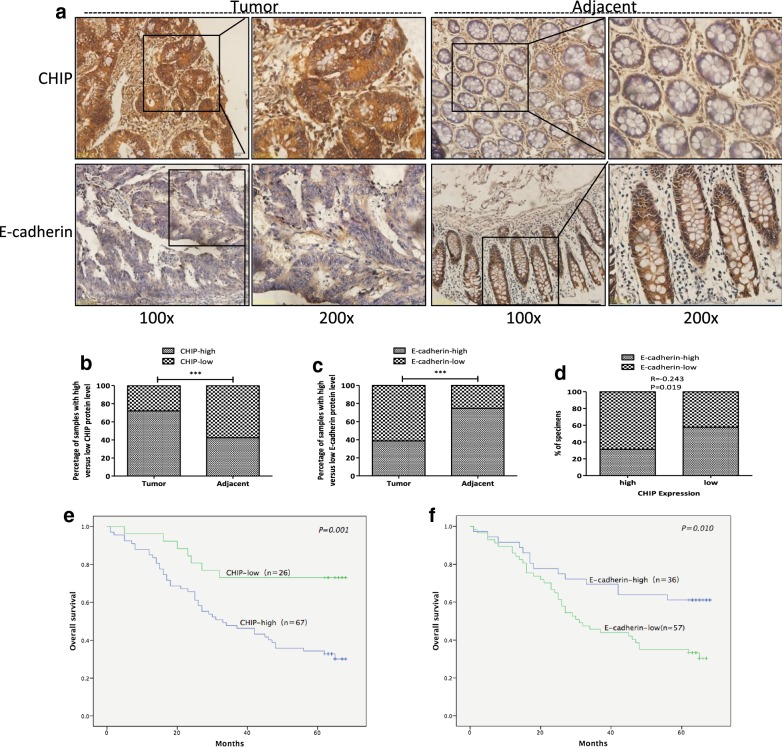
Clinical significance of CHIP and E-cadherin expression in CRC tissues. **a** Representative images of CHIP and E-cadherin expression in CRC and adjacent non-neoplastic tissues were shown by IHC. Tumor represented CRC cancer tissues; Adjacent represented the non-neoplastic tissues; magnification ×100 and ×200). Higher expression of CHIP was observed in the membrane and cytoplasm of CRC cells compared to that of the adjacent counterparts. E-cadherin was weekly positive or negative in the membrane and the cytoplasm of CRC cells. However, the majority of the adjacent counterparts cells were E-cadherin positive. **b**, **c** The stacked bars indicate the percentages of CRC samples with high and low CHIP and E-cadherin expression levels relative to the total number of tissues (χ^2^ test, ***P < 0.001). **d** The stacked bars indicate the percentages of CRC samples with high and low E-cadherin expression levels relative to the total number of CHIP-high and CHIP-low sections, respectively (Spearman’s test, ***P < 0.001). **e** Kaplan–Meier curves that depict the 5-year overall survival according to the CHIP expression in patients with CRC (n = 93, *P *= 0.001). CHIP-low represented the CHIP low expression group; CHIP-high represented the CHIP high expression group. The differences of the overall survival between these two groups were determined using a log-rank test. **f** Kaplan–Meier curves that depict the 5-year overall survival according to the CHIP expression in patients with CRC (n = 93, *P *= 0.010). E-cadherin-low represented the E-cadherin low expression group; E-cadherin-high represented the E-cadherin high expression group. The difference of the overall survival between these two groups was determined using a log-rank test

A tissue microarray of 93 CRC patients with paired adjacent counterparts was evaluated for the expression of CHIP and E-cadherin protein by IHC. CHIP was predominantly expressed on the membrane and in the cytoplasm of the cancer cells compared to that in the adjacent parts. While E-cadherin was predominantly expressed on the membrane and in the cytoplasm of the adjacent non-neoplastic cells compared to that in cancer part. Representative images of CHIP and E-cadherin expression in cancer and adjacent non-neoplastic tissues were shown in Fig. 5a.

As shown in Fig. 5b, significantly increased CHIP expression (CHIP-high) was found in 72.04% (67/93) of CRC samples, while higher CHIP expression was only observed in 42.5% (37/87) of the paired adjacent non-neoplastic tissues. CHIP expression was much higher in cancer tissues when compared with that in adjacent counterparts (*P *< 0.001). Intriguingly, significantly decreased E-cadherin expression (E-cadherin-low) was found in 61.29% (57/93) of CRC samples, while decreased E-cadherin expression was only observed in 25.3% (22/87) of the paired adjacent non-neoplastic tissues. E-cadherin expression was much lower in cancer tissues when compared with that in adjacent counterparts (*P *< 0.001, Fig. 5c). Also, Spearman rank correlation analysis showed that the expression of CHIP and E-cadherin expression was negatively correlated (r = − 0.243, *P *= 0.019, Fig. 5d).

As shown in Table 1, the expression of CHIP was positively correlated with depth of tumor invasion (*P *= 0.039), lymph nodes invasion (*P *= 0.003), and TNM stage (*P *= 0.003). The CHIP expression was not significantly correlated with age, gender, site, differentiation or metastasis stage. E-cadherin expression was negatively correlated with depth of tumor invasion (*P *= 0.048), lymph nodes invasion (*P *= 0.043), and TNM stage (*P *= 0.020). There was no significant correlation of E-cadherin expression with age, gender, site, differentiation or metastasis stage. Kaplan–Meier analysis revealed that CRC patients with high CHIP expression were significantly correlated with a poorer overall survival than those with low CHIP expression (*P *= 0.001) (Fig. 5e). Low E-cadherin expression was evidently correlated with poorer overall survival of CRC patients than high E-cadherin expression (*P *= 0.010) (Fig. 5f).

**Table 1:** The correlation of CHIP and E-cadherin expression with the clinical characteristics of CRC patients

Characteristics	Nr. of patients (total: 93)	CHIP expression			E-cadherin expression		
		High	Low	*P* value	High	Low	*P* value
		Nr. (N = 67)	Nr. (N = 26)		Nr. (N = 36)	Nr. (N = 57)	
Age (years)	68 (22–93)			0.231			0.210
≤ 60	34	22	12		16	18	
> 60	59	45	14		20	39	
Gender				0.830			0.422
Male	52	37	15		22	30	
Female	41	30	11		14	27	
Site				0.708			0.099
Right	34	25	9		10	24	
Left	53	37	16		25	28	
Unknown	6	5	1		1	5	
Differentiation				0.456			0.32
Well	2	2	0		2	0	
Moderate	73	53	20		28	45	
Poor	18	12	6		6	12	
Depth of tumor invasion				0.039*			0.048*
T1	0	0	0		0	0	
T2	5	2	3		3	2	
T3	64	44	20		27	37	
T4	16	14	2		3	13	
Unknown	8	7	1		3	5	
Lymph nodes invasion				0.003**			0.043*
N0	51	33	18		24	27	
N1	28	25	3		7	21	
N2	9	8	0		2	6	
Unknown	6	1	5		3	3	
Metastasis				0.481			0.960
M0	89	63	26		35	54	
M1	4	4	0		1	3	
TNM stage				0.003**			0.020*
I	4	2	2		3	1	
II	41	26	15		19	22	
III	35	31	4		9	26	
IV	4	4	0		1	3	
Unknown	9	4	5		4	5	

As shown in Table 2, tumor differentiation, lymph node metastasis, TNM stage, CHIP and E-cadherin expression showed statistical significances in the univariate survival analysis, respectively (*P *< 0.01). Multivariate Cox regression analysis indicated that CHIP expression and tumor differentiation had statistical significances, with hazard ratio of 7.163 for CHIP (95% CI 2.188–23.453, *P *= 0.001) and 3.030 for tumor differentiation (95% CI 1.485–6.180, *P *= 0.002), indicating the CHIP expression and tumor differentiation were independent prognostic factors in the development of CRC.

**Table 2:** Univariate and multivariate analyses of OS in 74 patients with CRC patients

Characteristics	Univariate analysis		Multivariate analysis	
	HR (95%) CI	*P* value	HR (95%) CI	*P* value
Age (≥ 60 vs. < 60)	1.504 (0.826–2.736)	0.182	–	–
Gender (male vs. female)	0.825 (0.480–1.416)	0.485	–	–
Differentiation (poor vs. well, moderate)	2.948 (1.618–5.369)	< 0.001***	3.030 (1.485–6.180)	0.002**
Site (right vs. left)	0.729 (0.410–1.296)	0.281	–	–
Depth of tumor invasion (T4 vs. T2, T3)	1.567 (0.801–3.066)	0.190	–	–
Lymph nodes invasion (N1, N2 vs. N0)	2.740 (1.557–4.820)	< 0.001***	–	–
Metastasis (M1 vs. M0)	2.380 (0.850–6.667)	0.099	–	–
TNM stage (III, IV vs. I, II)	2.927 (1.640–5.222)	< 0.001***	–	–
CHIP expression (high vs. low)	3.465 (1.561–7.693)	0.002**	7.163 (2.188–23.453)	0.001***
E-cadherin expression (high vs. low)	0.459 (0.249–0.847)	0.013*	–	–

## Discussion

According to most reports, CHIP behaves as a tumor suppressor in many malignancies, such as human pancreatic cancer cells, lung cancer, gastric cancer, and breast cancer [23, 28, 31, 37]. However, accumulating evidence have also implicated that CHIP can serve as an oncogene in several other human malignancies [32, 35, 38]. The pathogenic mechanisms of CHIP in human malignancies remain controversial. This inconsistency might be contributed to the various targeting proteins of CHIP in different cellular context. In human glioma, cell proliferation is suppressed in CHIP silencing U251 and U87 glioma cells, while is promoted in CHIP-overexpressing cells [32]. CHIP functions as an oncogene and supports cell growth of thyroid cancer cells through activation of MAPK and AKT pathways [35]. Consistently, our data indicated that cell growth was inhibited in the CHIP-silencing DLD-1 cells. CHIP silencing led to suppressed cell proliferation and G0–G1 arrest of CRC cells, accompanied by the inhibited MAPK and AKT signaling activities, and downregulated cyclinD1. CHIP overexpression exerted no obvious influence on the cell proliferation and cell cycle of DLD-1 cells. The MAPK and AKT pathways can be uncontrollably activated by mutant *KRAS*, leading to promoted cell proliferation, migration and invasion in tumor cells [39–41]. Considering the dominant role of the *KRAS* mutation in DLD-1 cells, overexpression of CHIP may be not able to influence the cell growth of DLD-1.

Previous reports reveal that CHIP negatively regulates the NF-κB signaling by promoting ubiquitination and degradation of p65 in CRC HCT-116 cell line [24]. CHIP inhibits the NF-κB-mediated cell invasion via down-regulating TRAF2 in breast cancer [23]. Unlike the previous findings, our results showed that CHIP didn’t affect the activities of the canonical or non-canonical NF-κB signaling pathways.

Invasion and metastasis are primary processes for the cancer progression. In breast cancer, CHIP promotes the MDA-MB231 cell migration through down-regulating Pfn1 [42]. In the present study, we demonstrated that CHIP promoted cell migration and invasion in CRC cell lines, via x-Celligence monitoring, transwell assay, wound healing assay, and mesentery metastasis model in vivo. CHIP silencing attenuated the DLD-1 cell migratory and invasive potential in vitro and metastasis in vivo. Conversely, cell migratory and invasive potential in vitro and metastasis in vivo were enhanced dramatically in CHIP-overexpressing DLD-1 cells. The promoted cell migratory and invasive abilities were also verified in CHIP-overexpressing HT-29 cells, suggesting that the function of CHIP is not specific for DLD-1 cells only.

**Fig. 6:**
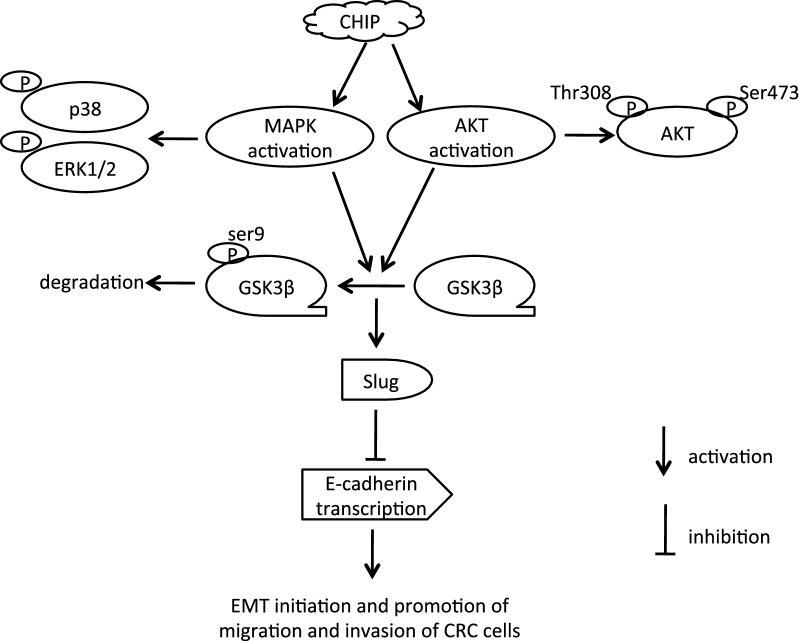
Schematic representation of CHIP in the modulation of EMT in CRC. CHIP activated the MAPK and AKT signaling pathway, which resulted in the inactivation of GSK-3β by phosphorylation at ser9. Consequently, the GSK-3β inactivation upregulated Slug and resulted in the decreased expression of E-cadherin by transcriptional repression

Suppression or loss of E-cadherin is critical for EMT and contributes to the progression and metastasis of a variety of malignancies [11, 12]. E-cadherin can be deregulated by several mechanisms, including genetic mutations, epigenetic silencing, and endocytosis and proteolytic processing in carcinoma [43, 44]. Promoter hypermethylation and direct binding of specific transcriptional repressors to the promoter of E-cadherin are two types of epigenetic silencing mechanisms in E-cadherin [45, 46]. The zinc-finger transcription factors, Snail and Slug, are known to bind to the E-box element in the *E*-*cadherin* proximal promoter and transcriptionally repress the E-cadherin expression [13, 14, 16]. In the present study, we found that E-cadherin expression was conspicuously elevated in the CHIP-silencing cells, while was obviously downregulated in the CHIP-overexpressing cells at both the transcription and translational levels. Furthermore, E-cadherin expression was also confirmed to be downregulated in hCHIP cells by the LC–MS/MS analysis. In addition, the expression of Slug protein was suppressed in the CHIP-silencing cells while upregulated in the CHIP-overexpressing cells. These results suggest that E-cadherin is transcriptionally downregulated by Slug in the CHIP-overexpressing cells. It is the first time we identified that the increase of cell migratory and invasive potential by CHIP was mediated through EMT via downregulation of E-cadherin in CRC. Previous data implicated that GSK-3β, inactivated by the activation of MAPK and AKT signaling pathway, could promote the degradation of Slug, and subsequently triggers EMT in lung cancer cells [21]. In the current study, we found that CHIP silencing in DLD-1 cells caused deduced expression of Slug, coupled with the GSK-3β activation by the downregulation of MAPK and AKT signaling (Fig. 1e). On the contrary, overexpression of CHIP led to the increased expression of Slug, accompanying with GSK-3β inactivation by the upregulation of MAPK and AKT signaling (Fig. 2e). Collectively, CHIP probably activates the MAPK and AKT signaling pathway, which resulted in the inactivation of GSK-3β by phosphorylation at ser9. Consequently, the GSK-3β inactivation upregulated Slug and resulted in the decreased expression of E-cadherin in DLD-1 cells. The enhanced migration and invasion abilities were predominantly due to the occurrence of EMT regulated by the CHIP-MAPK/AKT-GSK-3β-Slug-E-cadherin signaling pathways (Fig. 6).

Comprehensive proteomic analysis by LC–MS/MS analysis revealed that 790 proteins with a cutoff value of 1.5-fold change were differentially expressed in CHIP-overexpressing cells when compared to those in ctrl cells, including 414 upregulated proteins and 376 downregulated proteins. Disease Enrichment analysis showed that 9 of the 790 differentially expressed proteins were correlated to the CRC disease, including E-cadherin. E-cadherin was also among the differentially expressed proteins correlated to cell–cell adhesion based on GO enrichment analysis and the cell–cell junction KEGG pathway analysis.

In the present study, we found that CHIP expression was positively correlated with depth of tumor invasion, lymph nodes invasion, and TNM stage. Multivariate Cox regression analysis revealed that CHIP was an independent prognostic factor in CRC. In consistent with in vitro results, we showed that CHIP and E-cadherin expression was negatively correlated. There was a lower E-cadherin level in CRC samples compared to that in the adjacent non-neoplastic tissues. The expression of E-cadherin was negatively correlated with depth of tumor invasion, lymph nodes invasion and TNM stage. Higher CHIP and lower E-cadherin expression were significantly correlated with a poorer overall survival of CRC patients. Mounting evidence indicates that the suppressed expression of E-cadherin have been considered as a valuable prognostic factor and correlated with the worse clinical characteristics and poor prognosis of CRC [10, 47–50].

## Conclusions

In conclusion, we considered that CHIP is a novel potential unfavorable independent predictive biomarker for CRC. Unlike the findings in several human malignancies, we identified that CHIP functions as an oncogene in CRC. CHIP interferes with a diverse aspect of cellular behaviors in CRC cells, such as cell proliferation, migration, and invasion, both in vitro and in vivo. The affected migration and invasion abilities were predominantly due to the occurrence of EMT through the CHIP-MAPK/AKT-GSK-3β-Slug-E-cadherin pathways.

## Additional files

**Additional file 1.** The primer sequences of the *CHIP*, *CDH1*, *EpCAM*, *CK8*, *CK18* and *β-actin* gene.

**Additional file 2.** CHIP expression in individual colorectal cancer cell lines. (a) qRT-PCR analysis of the mRNA expression of *CHIP* in HT-29, DLD-1, COLO320DM, and CaCO2 cell lines. *β-actin* normalized gene expression was displayed as an internal control. Independent experiments were displayed at least three times. Error bars were calculated as *SD* of the mean. (b) Western blotting analysis of the protein expression level of CHIP in the whole cell extract of HT-29, DLD-1, COLO320DM, and CaCO_2_ cell lines. Actin was used as a loading control.

**Additional file 3.** Establishment of CHIP-sliencing and CHIP-overexpressing DLD-1 cells. (a) qRT-PCR analysis of the mRNA expression of *CHIP* between the two established siCHIP and sictrl cell lines. *β-actin* normalized gene expression, measured in triplicates was displayed. Significant differences were indicated (Student’s t-test, ***P < 0.001). (b) The protein expression of *CHIP* in the whole cell extract of siCHIP and sictrl cell lines was analyzed by western blotting. The level of each protein was normalized against Actin. (c) qRT-PCR analysis of the mRNA expression of CHIP between the two established hCHIP and ctrl cells. *β-actin* normalized gene expression, measured in triplicates is displayed. Significant differences were indicated (Student’s t-test, ***P < 0.001). (d) The protein expression of CHIP in the whole cell extract of hCHIP and ctrl cells was analyzed by western blotting. The level of each protein was normalized against Actin.

**Additional file 4.** CHIP overexpression promoted the migration and invasion of HT-29 Cells. (a) qRT-PCR analysis of the mRNA expression of *CHIP* between the two established hCHIP and ctrl cells. *β-actin* normalized gene expression, measured in triplicates was displayed. Significant differences were indicated (Student’s t-test, ***P < 0.001). (b) The protein expression of CHIP in hCHIP and ctrl cells was analyzed by western blotting. The level of each protein was normalized against Actin. (c) The cell growth curves of the hCHIP and ctrl cells were detected by x-Celligence system. E-plate was plated with 10,000 cells/well and the cell growth was continuous monitored for 96 h. (d) Cell proliferation of the hCHIP and ctrl cells were determined by the Brdu proliferation assay. 96-well plate was plated with 10,000 cells/well and was added 10 μl buffer after cultured for 24, 48 and 72 h. OD450 was measured using spectrophotometer microplate reader. (e, f) CHIP enhanced the ability of migration (e) and invasion (f) of HT-29 cells measured by transwell assay. 40,000 HT-29 stable CHIP-overexpression and control cells were added to the upper inserts coated with or without Matrigel. The number of migrated and invasive cells were fixed, stained, photographed, and compared with the control group. Each bar represents the mean ± SD. ***P < 0.001. All images were representative of at least three independent experiments with similar findings, ×200 magnification.

## Abbreviations

CHIP: the carboxyl terminus of Hsc70-interacting protein
CRC: colorectal cancer
LC–MS/MS: liquid chromatography–tandem mass spectrometry
EMT: epithelial–mesenchymal transition
CK8/18: cytokeratin 8/18
EpCAM: epithelial cell adhesion molecular
MAPK: mitogen-activated protein kinase
GSK-3β: glycogen synthase kinase 3β
qRT-PCR: quantitative real-time RT-PCR
STUB1: STIP1 homology and U-box containing protein 1
TPR: tetratricopeptide repeat
TRAF2: TNF receptor-associated factor 2
NF-κB: nuclear factor kappa-light-chain-enhancer of activated B cells
cErbB2/Neu: receptor tyrosine-protein kinase erbB-2
EGFR: epidermal growth factor receptor
PRMT5: protein arginine methyltransferase 5
ESCC: esophageal squamous cell carcinoma
OD: optical density
SD: standard deviation
