# Supplementary material for: CHIP functions as an oncogene by promoting colorectal cancer metastasis via activation of MAPK and AKT signaling and suppression of E-cadherin
Source: J Transl Med. 2018 Jun 19;16:169. doi: 10.1186/s12967-018-1540-5 (PMC6008917; doi:10.1186/s12967-018-1540-5)
Supplement: Supplementary file 1 — Additional file 1. The primer sequences of the CHIP, CDH1, EpCAM, CK8, CK18 and β-actin gene. [file 12967_2018_1540_MOESM1_ESM.docx]

| **Primer name** | **NCBI Reference sequence** | **Sequences** | **Amplicon size** |
| --- | --- | --- | --- |
| ***CHIP*** | NM_005861 | sense: 5’-GCCAAGGAGCAGCGGCTGAA-3’ | 155 bp |
|  |  | antisense: 5’-CTCTCACGCTCCGCGGCAAT-3’ |  |
| ***CDH1*** | NM_001317185.1 | sense: 5’-GCTGGACCGAGAGAGTTTCC-3’ | 179 bp |
|  |  | antisense: 5’-CGACGTTAGCCTCGTTCTCA-3’ |  |
| ***EpCAM*** | NM_002354.2 | sense: 5’- CTCAGGAAGAATGTGTCTGTGA-3’ | 161 bp |
|  |  | antisense: 5’-TGAGCCATTCATTTCTGCCT-3’ |  |
| ***CK8*** | NM_002273.3 | sense: 5’-ACCAGGAGCTGATGAACGTC-3’ | 151 bp |
|  |  | antisense: 5’-CTCAGACCACCTGCATAGCC-3’ |  |
| ***CK18*** | NM_000224.2 | sense: 5’-GAGGGCTCAGATCTTCGCAA -3’ | 191 bp |
|  |  | antisense: 5’-CCAGCTGCAGTCGTGTGATA -3’ |  |
| ***β-actin*** | NM_001101 | sense: 5’-GCTACGAGCTGCCTGACGG -3’ | 174 bp |
|  |  | antisense: 5’-TGTTGGCGTACAGGTCTTTGC -3’ |  |

**Additional file 1. The primer sequences of the *CHIP*, *CDH1*, *EpCAM*, *CK8*, *CK18* and *β-actin* gene**
