# Supplementary figures and images for: CHIP functions as an oncogene by promoting colorectal cancer metastasis via activation of MAPK and AKT signaling and suppression of E-cadherin
Source: J Transl Med. 2018 Jun 19;16:169. doi: 10.1186/s12967-018-1540-5 (PMC6008917; doi:10.1186/s12967-018-1540-5)

a

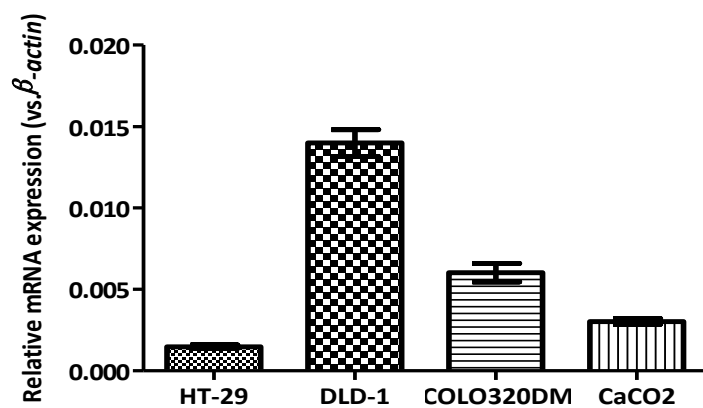

b

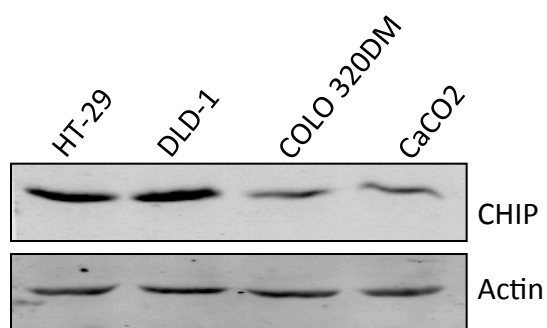

Additional file 2

Supplement: Supplementary file 2 — Additional file 2. CHIP expression in individual colorectal cancer cell lines. (a) qRT-PCR analysis of the mRNA expression of CHIP in HT-29, DLD-1, COLO320DM, and CaCO2 cell lines. β-actin normalized gene expression was displayed as an internal control. Independent experiments were displayed at least three times. Error bars were calculated as SD of the mean. (b) Western blotting analysis of the protein expression level of CHIP in the whole cell extract of HT-29, DLD-1, COLO320DM, and CaCO2 cell lines. Actin was used as a loading control. [file 12967_2018_1540_MOESM2_ESM.pdf]

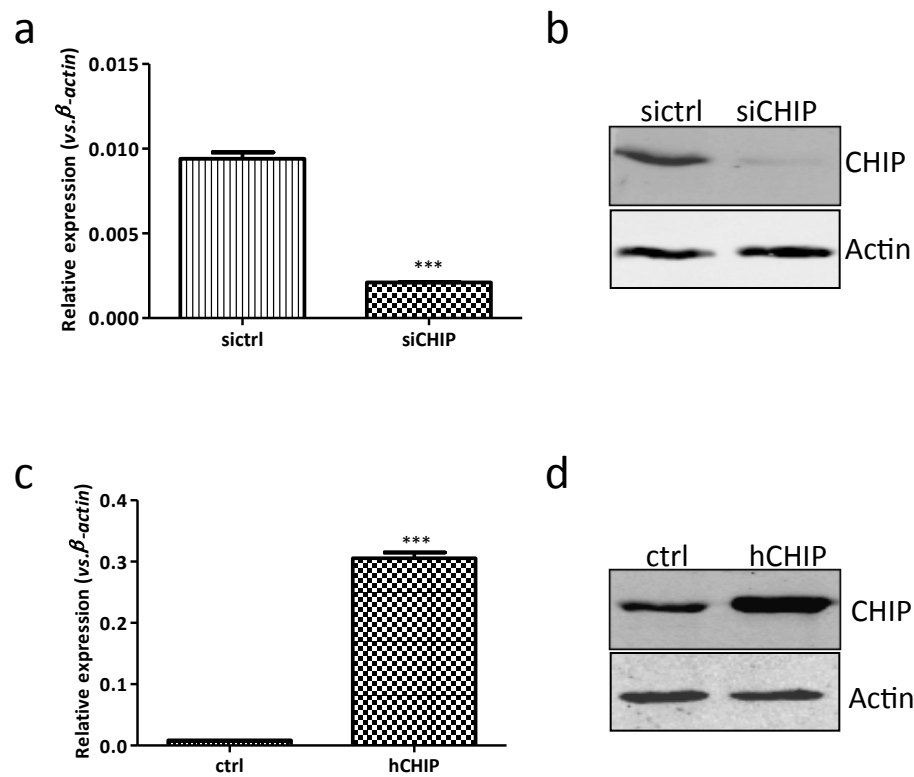

Additional file 3

Supplement: Supplementary file 3 — Additional file 3. Establishment of CHIP-sliencing and CHIP-overexpressing DLD-1 cells. (a) qRT-PCR analysis of the mRNA expression of CHIP between the two established siCHIP and sictrl cell lines. β-actin normalized gene expression, measured in triplicates was displayed. Significant differences were indicated (Student’s t-test, ***P < 0.001). (b) The protein expression of CHIP in the whole cell extract of siCHIP and sictrl cell lines was analyzed by western blotting. The level of each protein was normalized against Actin. (c) qRT-PCR analysis of the mRNA expression of CHIP between the two established hCHIP and ctrl cells. β-actin normalized gene expression, measured in triplicates is displayed. Significant differences were indicated (Student’s t-test, ***P < 0.001). (d) The protein expression of CHIP in the whole cell extract of hCHIP and ctrl cells was analyzed by western blotting. The level of each protein was normalized against Actin. [file 12967_2018_1540_MOESM3_ESM.pdf]

a

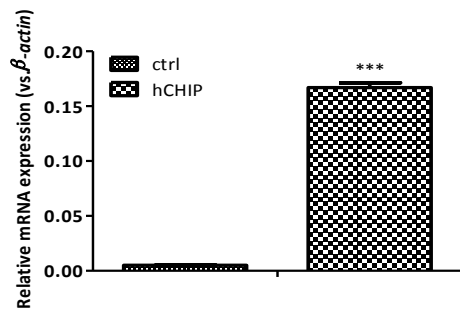

b

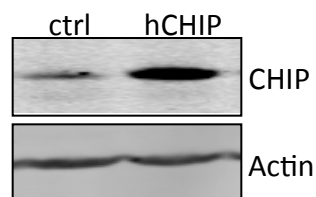

c

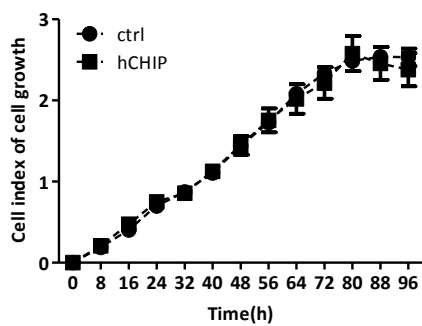

d

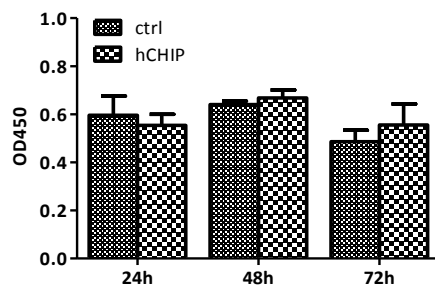

e

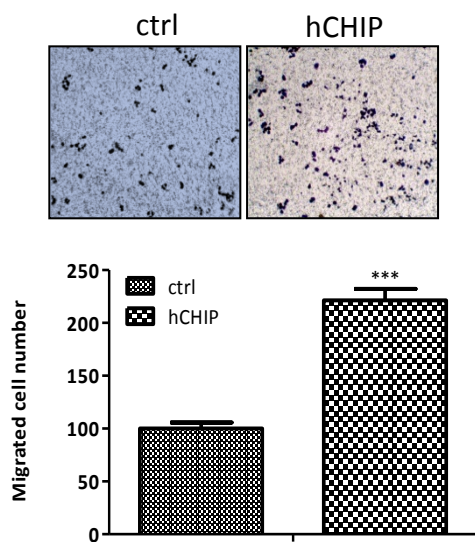

f

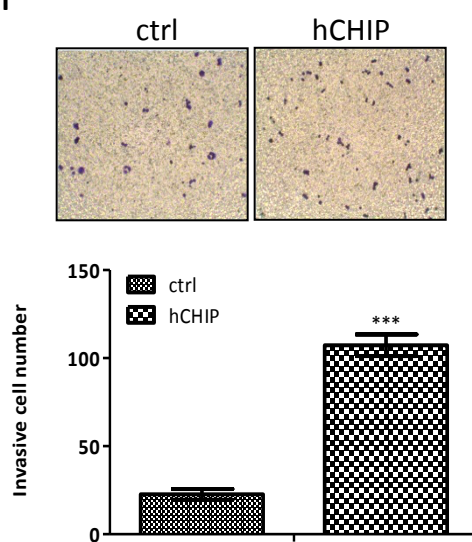

Supplement: Supplementary file 4 — Additional file 4. CHIP overexpression promoted the migration and invasion of HT-29 Cells. (a) qRT-PCR analysis of the mRNA expression of CHIP between the two established hCHIP and ctrl cells. β-actin normalized gene expression, measured in triplicates was displayed. Significant differences were indicated (Student’s t-test, ***P < 0.001). (b) The protein expression of CHIP in hCHIP and ctrl cells was analyzed by western blotting. The level of each protein was normalized against Actin. (c) The cell growth curves of the hCHIP and ctrl cells were detected by x-Celligence system. E-plate was plated with 10,000 cells/well and the cell growth was continuous monitored for 96 h. (d) Cell proliferation of the hCHIP and ctrl cells were determined by the Brdu proliferation assay. 96-well plate was plated with 10,000 cells/well and was added 10 μl buffer after cultured for 24, 48 and 72 h. OD450 was measured using spectrophotometer microplate reader. (e, f) CHIP enhanced the ability of migration (e) and invasion (f) of HT-29 cells measured by transwell assay. 40,000 HT-29 stable CHIP-overexpression and control cells were added to the upper inserts coated with or without Matrigel. The number of migrated and invasive cells were fixed, stained, photographed, and compared with the control group. Each bar represents the mean ± SD. ***P < 0.001. All images were representative of at least three independent experiments with similar findings, ×200 magnification. [file 12967_2018_1540_MOESM4_ESM.pdf]
